# Abnormal splicing in the N‐terminal variable region of cardiac troponin T impairs systolic function of the heart with preserved Frank‐Starling compensation

**DOI:** 10.14814/phy2.12139

**Published:** 2014-09-04

**Authors:** Han‐Zhong Feng, Guozhen Chen, Changlong Nan, Xupei Huang, Jian‐Ping Jin

**Affiliations:** 1 Department of Physiology, Wayne State University School of Medicine, Detroit, Michigan; 2 Charles E. Schmidt College of Medicine, Florida Atlantic University, Boca Raton, Florida

**Keywords:** Abnormal splicing, cardiac function, muscle contraction, N‐terminal variable region, troponin T isoforms

## Abstract

Abnormal splice‐out of the exon 7‐encoded segment in the N‐terminal variable region of cardiac troponin T (cTnT‐ΔE7) was found in turkeys and, together with the inclusion of embryonic exon (eTnT), in adult dogs with a correlation with dilated cardiomyopathy. Overexpression of these cTnT variants in transgenic mouse hearts significantly decreased cardiac function. To further investigate the functional effect of cTnT‐ΔE7 or ΔE7+eTnT in vivo under systemic regulation, echocardiography was carried out in single and double‐transgenic mice. No atrial enlargement, ventricular hypertrophy or dilation was detected in the hearts of 2‐month‐old cTnT‐ΔE7 and ΔE7+eTnT mice in comparison to wild‐type controls, indicating a compensated state. However, left ventricular fractional shortening and ejection fraction were decreased in ΔE7 and ΔE7+eTnT mice, and the response to isoproterenol was lower in ΔE7+eTnT mice. Left ventricular outflow tract velocity and gradient were decreased in the transgenic mouse hearts, indicating decreased systolic function. Ex vivo working heart function showed that high afterload or low preload resulted in more severe decreases in the systolic function and energetic efficiency of cTnT‐ΔE7 and ΔE7+eTnT hearts. On the other hand, increases in preload demonstrated preserved Frank‐Starling responses and minimized the loss of cardiac function and efficiency. The data demonstrate that the N‐terminal variable region of cardiac TnT regulates systolic function of the heart.

## Abstract (precis)

Using transgenic mouse models expressing myopathic splicing variants of cardiac troponin T, we demonstrated that abnormality in the N‐terminal variable region of troponin T selectively affects the systolic function of the heart, whereas the Frank‐Starling response is preserved.

## Introduction

The contraction and relaxation of skeletal and cardiac muscles are regulated by intracellular Ca^2+^ via troponin in the sarcomeric thin filament (Gordon et al. 2000). The troponin complex consists of three protein subunits, troponin C (TnC), troponin I (TnI) and troponin T (TnT). Troponin T coordinates the structure and function of troponin complex and is the thin filament anchoring molecule (Perry 1998). The N‐terminal segment of TnT is a hypervariable region that differs among muscle type‐specific isoforms and regulated via alternative RNA splicing during development and adaptation (Wang and Jin 1998; Jin and Root 2000; Jin et al. 2000; Biesiadecki and Jin 2002; Biesiadecki et al. 2002, 2007; Feng et al. 2008).

Functional differences have been found between TnT isoforms and splice forms differing in the N‐terminal variable region. A larger to smaller, more acidic to less acidic, switch occurs in the expression of both cardiac and fast skeletal muscle TnT during perinatal development (Cooper and Ordahl 1985; Jin and Lin 1989; Jin et al. 1992, 1996; Wang and Jin 1997). Biochemical and contractility studies have demonstrated functional differences between embryonic and adult cardiac TnT (Jin and Lin 1989; Gomes et al. 2004).

Aberrant splicing of exon 4 that encodes 4–5 amino acids in the N‐terminal variable region of cardiac TnT has been found in failing human hearts (Anderson et al. 1995; Mesnard‐Rouiller et al. 1997), diabetic rat hearts (Akella et al. 1995), and hypertrophic rat hearts (McConnell et al. 1998). Abnormal omission of exon 8 occurs in turkey hearts with inherited dilated cardiomyopathy (Biesiadecki and Jin 2002). The same exon (exon 7 in mammalian cardiac TnT) was abnormally spliced out in dog hearts with dilated cardiomyopathy (cTnT‐ΔΕ7) (Biesiadecki et al. 2002). This N‐terminal region coding exon is constitutively included in normal cardiac TnT (Jin et al. 2008). Its aberrant splice‐out in dilated turkey and dog cardiomyopathies indicates a causal relationship to the pathogenesis. In addition to the splice‐out of exon 7, dilated cardiomyopathy dog hearts showed abnormal inclusion of the embryonic exon 5 in cardiac TnT (eTnT) in the adult cardiac muscle (Biesiadecki et al. 2002).

The coexistence of two or more cTnT variants resulting in split myofilament Ca^2+^ sensitivity (Biesiadecki et al. 2002; Gomes et al. 2004) would cause a temporally desynchronized myofilament response to the rising of intracellular Ca^2+^ during the activation of contraction. After the alternative splicing‐generated cTnT isoform switch during perinatal heart development (Jin and Lin 1988), a single form of cardiac troponin is present in adult cardiac muscle of human and most other vertebrates, corresponding to the notion that a uniformed Ca^2+^ activation of the thin filaments generates a synchronized contraction. Our previous studies have demonstrated that the coexistence of functionally distinct TnT isoforms (Huang et al. 1999) or N‐terminal splicing variants (Huang et al. 2008; Feng and Jin 2010; Wei et al. 2010) with altered Ca^2+^ activation of force production resulted in decreased pumping function and energetic efficiency. Further evidence from chronic coexistence of two TnT isoforms in adult transgenic mouse heart also showed decreased contractile and Ca^2+^ transient kinetics in cardiomyocytes (Yu et al. 2012).

The present study investigated the pathogenic phenotype of the abnormally spliced variants cardiac TnT in vivo in transgenic mice overexpressing cTnT‐ΔΕ7 or ΔΕ7 + eTnT at young age prior to the development of anatomical cardiomyopathy using echocardiography under systemic neurohumoral regulation, followed by ex vivo working heart studies on isolated organ function. In addition to detecting early changes in cardiac function, the results showed that the N‐terminal abnormality of cardiac TnT impaired systolic function and energetic efficiency, whereas Frank‐Starling response of the heart was preserved to compensate cardiac function, providing valuable insights into the structure‐function relationship of troponin and the pathogenic mechanism for cTnT N‐terminal abnormality to generate dilated cardiomyopathy.

## Methods

### Ethical approval

All animal protocols are approved by the Institutional Animal Care and Use Committees of Wayne State University and Florida Atlantic University, and conformed to the guidelines of the National Institutes of Health Guide for the Care and Use of Laboratory Animals.

### Transgenic mice

**Figure 1.**
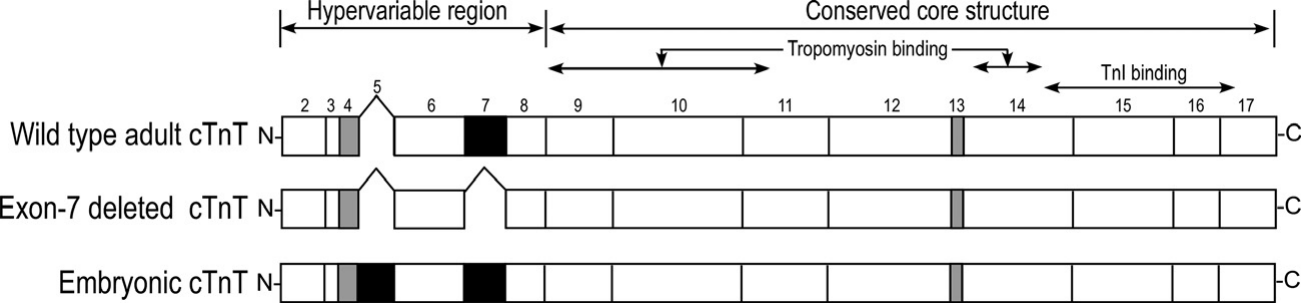
Abnormal splicing variants of cardiac TnT. The primary structural alignment shows the N‐terminal splicing patterns of wild‐type adult cardiac TnT, exon 7‐deleted cardiac TnT and embryonic cardiac TnT. The developmentally regulated exon 5 and abnormally deleted exon 7 are shown as solid black boxes. Exons 4 and 13 that are also alternatively spliced in normal mouse cardiac TnT are shown as gray boxes. The tropomyosin‐ and TnI‐binding sites (Jin and Chong 2010; Wei and Jin 2011) are outlined.

The two transgenic mouse lines used in this study have been generated in previous studies to postnatally overexpress cTnT‐ΔΕ7 or eTnT (Fig. 1) under the control of an *α*‐myosin heavy chain (MHC) promoter in C57BL/6 strain (Biesiadecki et al. 2002).

Double‐transgenic mice were generated by crossing the cTnT‐ΔΕ7 and eTnT lines to combine the overexpression of ΔΕ7+eTnT. Genotyping of the transgenic mice was carried out using PCR on tail biopsies as described previously (Huang et al. 1999). We have previously demonstrated that the total level of myocardial TnT remained normal in the single and double‐transgenic mouse lines (Feng and Jin 2010), which provides effective replacement models for functional studies.

Mice were maintained on a 12:12‐h light‐dark cycle (6:00 am/6:00 pm) and fed with standard pellet diet. Two‐month‐old female mice were used for echocardiographic measurement and 3‐ to 5‐month‐old mice of both sexes were used for ex vivo working heart studies.

### Echocardiography

Echocardiography studies were performed using a Vevo 770 high‐resolution in vivo imaging system (VisualSonics, Toronto, ON, Canada) as described previously (Li et al. 2013). To exclude experimental bias, all measurements were done by an examiner blinded to the genotypes. The mice were anesthetized with 1.2% isoflurane and placed on a heating pad to maintain body temperature at 37°C. Hair on the precordial region was removed with Nair lotion hair remover, and the region was covered with ultrasound transmission gel (Aquasonic, Parker Laboratory, Fairfield, NJ). Short‐axis images under the M‐mode were taken to view the left ventricle (LV) and right ventricle (RV) movements during diastole and systole, allowing us to measure the ventricular structure and dimension. Transmitral blood flow was measured with Pulse Doppler and diastolic mitral annular velocity was measured with Tissue Doppler. After measurement of baseline condition, isoproterenol (ISO) was administrated (0.2 mg kg^−1^ body weight, i.p.) and the same measurement was repeated for *β*‐adrenergic responses. All data and images were saved and analyzed with the Advanced Cardiovascular Package Software (VisualSonics) to evaluate cardiac function.

### Ex vivo working heart studies

Transgenic and wild‐type mouse hearts were examined in isolated ex vivo working heart preparations as previously described (Feng et al. 2008). Thirty minutes after injection of 100 Unit heparin i.p., mice were anesthetized with pentobarbital (100 mg kg^−1^ body weight, i.p.). Hearts were rapidly isolated and cannulated via aorta with a modified 18‐gauge needle to start Langendorff retrograde perfusion within 3 min after opening of the chest. A pressure sensor (MLT844 pressure transducer, Capto, Horten, Norway) was connected to the side arm of aortic cannular and placed at the level equivalent to the heart to measure aortic pressure. A 0.5 mL air bubble was introduced in the aortic trap to mimic in vivo arterial compliance. A pulmonary vein was then cannulated with a 16‐gauge needle for perfusion to the left atria in the working mode. The pulmonary artery trunk was connected to a beveled polyethylene‐25 tubing to collect the coronary effluent from the right ventricle. The coronary effluent was also measured for the O_2_ concentration by passing through an O_2_ sensor (Microelectrode). The apex was punctured using a 30‐gauge needle to make a path that allows the insertion of a 1.2‐Fr pressure–volume (P–V) catheter (Scisense, London, ON, Canada) into the LV. After all the cannulations were established, a water jacket was placed around the heart to maintain the surrounding temperature at 37°C before switching to left atrial perfusion to start the working mode.

The perfusion medium used was a modified Krebs‐Henseleit bicarbonate buffer equilibrated with 95% O_2_–5% CO_2_, containing 118 m·mol·L^−1^ NaCl, 4.7 m·mol·L^−1^ KCl, 1.2 m·mol·L^−1^ KH_2_PO_4_, 2.25 m·mol·L^−1^ MgSO_4_, 2.25 m·mol·L^−1^ CaCl_2_, 0.32 m·mol·L^−1^ EGTA, 2 m·mol·L^−1^ pyruvate, and 15 m·mol·L^−1^ D‐glucose. NaHCO_3_ was added to adjust the pH to 7.4 at 37°C. The perfusion buffer was filtered with a 0.45‐*μ*m filter membrane and not reused.

Baseline cardiac function was recorded at the standard preload of 10 mmHg and afterload of 55 mmHg (Barbato et al. 2005). Heart rate was controlled at 480 beats per min with supraventricular pacing using an isolated constant current stimulator (A365; World Precision Instruments, Sarasota, FL) through a pair of custom‐modified platinum wires attached to the surface of right atrium.

Aortic and coronary effluent volumes were recorded in real time by calibrated counting of drops of the outflow. Pressure and volume development data were collected at a sampling rate of 1 kHz with 100‐Hz filter using a Powerlab 16‐channel analog‐to‐digital interface and Chart 5.0 software (AD Instruments, Colorado Springs, CO). Preload response was tested by altering the height of preload perfusate reservoir at left atrial filling pressure of 5, 8, 10, 12.5, 15, and 20 mmHg. Afterload was adjusted by changing the height of effluent outlet equivalent to 55, 70, or 90 mmHg.

Immediately after functional measurements, LV muscle tissue was collected from each heart and stored at −80°C for Western blot verification of cardiac TnT contents.

### Time parameters of ex vivo working heart function

Left ventricular ejection time parameters were determined as previously described (Feng and Jin 2010). Briefly, the opening and closing of the aortic valve were identified by analyzing the traces of aortic pressure (AP). The first and highest peak of +dP/dt of AP, indicating the full opening of the aortic valve, was used as the beginning of the ejection. The lowest point of the AP curve at the end of the ejection phase, at which the dP/dt of AP = 0, was used as the time of aortic valve closing. The duration between these two points is the total LV ejection time. The rapid ejection phase was from the beginning of ejection to the peak of LV pressure (LVP). Isovolumetric contraction time was defined from the beginning of systole in LVP trace to the time of aortic valve opening. The isovolumetric relaxation time was measured from the end of ejection to the time when LVP decreased to the level of left atrial filling pressure that was equal to the preload.

### Calculation of cardiac efficiency

Cardiac efficiency was first evaluated by the ratio between LV ejection integral (the area under LVP curve during the ejection phase) and LV total integral (the total area under the LVP curve).

Left ventricular efficiency was further calculated from O_2_ consumption as previously described (Neely et al. 1967; Gauthier et al. 1998; Feng and Jin 2010): Cardiac efficiency (in %) = cardiac work/myocardial O_2_ consumption × 100. Myocardial O_2_ consumption was calculated from the difference between O_2_ concentrations in the perfusion influent and coronary effluent: O_2_ consumption (in mL·O_2_·min^−1^·g^−1^) = (PO_2a_−PO_2v_) × coronary flow × *c*/760 where PO_2a_ is PO_2_ in the perfusate (95%), PO_2v_ is PO_2_ in the coronary effluent, and *c* is the solubility coefficient for O_2_ in Krebs buffer (22.7 mL O_2_·atm^−1^·mL^−1^ at 37°C).

Pressure work and kinetic work were calculated as follows: Pressure work (in J·min^−1^·g^−1^) = cardiac output (in mL·min^−1^·g^−1^) × aortic pressure (in mmHg) × 1.33 ×10^−4^ J·mmHg^−1^·mL^−1^. Kinetic work (in J·min^−1^·g^−1^) =cardiac output (in mL·min^−1^·g^−1^) × [perfusate density (in g cm^−3^)/980 cm s^−2^] × *V*^2^ × 9.8 × 10^−3^ J·g^−1^·m^−1^·min^−1^·g^−1^ where *V* (in cm s^−1^) = [cardiac output (in mL min^−1^)/aortic cross‐sectional area (in cm^2^)] × [cycle time (in s)/ejection time (in s)] × (1/60). Myocardial O_2_ consumption was converted into joules per minute per gram using a conversion factor of 20.054 J mL^−1^ O_2_ consumed (Gauthier et al. 1998).

### SDS‐polyacrylamide gel electrophoresis (PAGE) and western blotting

Cardiac muscle from left ventricular free wall was rapidly isolated postmortem and homogenized in SDS‐PAGE sample buffer containing 2% SDS and 1% *β*‐mercaptoethanol, pH 8.8, using a high speed mechanical homogenizer to extract total proteins. The SDS‐PAGE samples were denatured by heating at 80°C for 5 min, centrifuged in a microcentrifuge to remove insoluble materials, and resolved on 14% SDS‐gel with an acrylamide:bisacrylamide ratio of 180:1 using a modified Laemmli buffer system in which both stacking and resolving gels were at pH 8.8. The protein bands resolved in the gel were stained with Coomassie Blue R‐250. Total protein in each lane was quantified by ImageJ software (National Institutes of Health, Bethesda, MD) for normalizing the amount of sample loading.

Duplicate SDS‐gels were transferred to nitrocellulose membrane using a Bio‐Rad semidry electrical transfer device at constant current of 5 mA cm^−2^ for 15 min. The blotted membranes were blocked in 1% bovine serum albumin (BSA) in Tris‐buffered saline (TBS, 150 m·molL^−1^ NaCl, 50 m·mol L^−1^ Tris, pH 7.5) with shaking at room temperature for 30 min. The blocked membrane was probed with an anti‐TnI monoclonal antibody (mAb) TnI‐1 (Jin et al. 2001) or an anti‐TnT mAb 2C8 that recognizes all TnT isoforms and splice forms (Jin and Chong 2010), both diluted in TBS containing 0.1% BSA, with gentle rocking at 4°C overnight. The membranes were then washed three times with TBS containing 0.5% Triton X‐100 and 0.05% SDS for 7 min each time and following with two times wash of TBS for 3 min of each time. After incubation with alkaline phosphatase‐labeled goat anti‐mouse IgG second antibody (Santa Cruz Biotechnology, Dallas, TX) at room temperature for 1 hour, membranes were washed again as above, and developed in 5‐bromo‐4‐chloro‐3‐indolyl phosphate/nitro blue tetrazolium substrate solution to visualize the cardiac TnI and cardiac TnT bands.

### Pro‐Q diamond phosphoprotein staining

To examine the effect of *β*‐adrenergic‐dependent phosphorylation of thin and thick filament proteins, Pro‐Q Diamond phosphoprotein staining (Invitrogen, Grand Island, NY) was employed following the manufacturer's instruction. Total cardiac muscle proteins were resolved on 14% SDS‐polyacrylamide gels as above. The SDS‐gel was fixed in 50% methanol and 10% acetic acid overnight with a change after the first 45 min. After washing in deionized water for three changes of 10 min each, the gel was stained with shaking in Pro‐Q Diamond reagent for 90 min in a dark box. Destaining was performed in 20% acetonitrile, 50 m·mol·L^−1^ sodium acetate, pH 4.0, for three changes of 30 min each in a dark box. The gel was then washed twice with deionized water for 5 min each in a dark box and scanned on a Typhoon 9410 fluorescence scanner (GE Healthcare, Wauwatosa, WI) with excitation at 532 nm and recording the emission at 560 nm to reveal phosphorylated proteins. The same gel was then stained with Coomassie Blue R‐250 to visualize the total protein profile.

### Data analysis

Data are presented as means ± SE or ± SD and statistical analysis was performed using Student's *t* test, or one‐way and two‐way ANOVA with a Fisher adjustment as noted in the table and figure legends.

## Results

### Impaired systolic function of cTnT‐ΔE7 and ΔE7+eTnT transgenic mouse hearts in vivo

**Figure 2.**
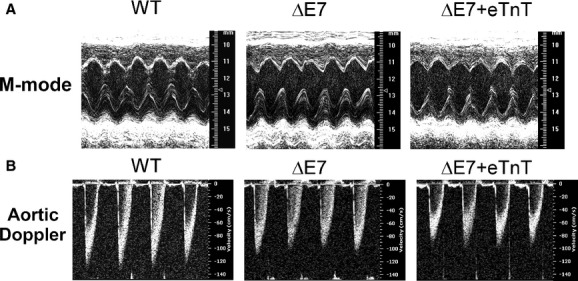
Decreased systolic function of transgenic mouse hearts detected in vivo using echocardiography. (A) M‐mode echocardiography showed motion (M) of the interfaces toward and away from the transducer along with the time axis. The results indicated a larger left ventricular end systolic dimension and volume in cTnT‐ΔΕ7 and ΔΕ7+eTnT than that in WT mice. (B) Doppler spectrum of the aorta showed that the velocity of blood flow during ejection was slower in cTnT‐ΔΕ7 and ΔΕ7+eTnT mice than that of WT group.

B‐mode and M‐mode echocardiography showed no atrial or ventricular enlargement or dilation in the transgenic mice in comparison with the wild‐type (WT) control at 2 months of age. Figure 2 shows representative images of the left ventricle under M‐mode and aortic blood flow under pulse wave Doppler for the three groups. Consistently normalized left ventricular mass values indicated no significant LV hypertrophy in the transgenic mice (Table 1). The results indicate no cardiac remodeling at anatomical level in the young mouse hearts and reflect a compensated state of these cardiomyopathy models.

**Table 1.** In vivo cardiac function measured with echocardiography.

Parameters	WT	ΔE7	ΔE7+eTnT
Body weight (g)	17.54 ± 0.36	17.31 ± 0.57	18.4 ± 0.61
Heart rate (bpm)	482 ± 4	483 ± 2	488 ± 4
LV end diastole			
IVS (mm)	0.82 ± 0.04	0.82 ± 0.03	0.81 ± 0.04
PW (mm)	0.74 ± 0.05	0.65 ± 0.06	0.63 ± 0.07
LVEDD (mm)	2.89 ± 0.11	3.07 ± 0.14	3.07 ± 0.07
LV Volume (*μ*L)	32.18 ± 3.00	37.66 ± 4.07	37.28 ± 2.15
LV End Systole			
IVS (mm)	1.31 ± 0.04	1.26 ± 0.06	1.20 ± 0.09
PW (mm)	1.30 ± 0.06	1.14 ± 0.07	1.10 ± 0.10
LVESD (mm)	1.44 ± 0.06	1.74 ± 0.07^*^	1.72 ± 0.06^*^
LV Volume (*μ*L)	5.61 ± 0.53	9.16 ± 0.92^*^	8.75 ± 0.70^*^
LV EF %	83.09 ± 1.08	75.44 ± 1.46^*^	76.21 ± 2.23^*^
LV FS %	50.51 ± 1.25	43.22 ± 1.27^*^	43.95 ± 2.30^*^
LV Mass Corrected, mg	53.57 ± 2.30	54.49 ± 2.41	53.03 ± 5.04
Mitral Pulsed Doppler			
E velocity (mm·s^−1^)	712.11 ± 5.82	704.24 ± 0.75	677.46 ± 7.79
A velocity (mm·s^−1^)	561.36 ± 17.46	456.36 ± 15.91	423.21 ± 23.95
E/A	1.27 ± 0.04	1.59 ± 0.11	1.63 ± 0.10
LVRT (ms)	16.27 ± 0.42	17.90 ± 1.20	17.70 ± 0.88
LVCT (ms)	9.20 ± 0.40	9.13 ± 0.50	11.02 ± 0.74^*^^#^
Mitral TDI			
E' velocity (mm·s^−1^)	30.44 ± 0.05	31.33 ± 0.80	29.31 ± 0.41
A' velocity (mm·s^−1^)	22.35 ± 0.25	23.40 ± 1.32	21.25 ± 0.37
E'/A'	1.37 ± 0.02	1.36 ± 0.05	1.40 ± 0.05
E/E'	23.39 ± 0.19	22.53 ± 0.53	23.12 ± 0.29
Aortic Pulsed Doppler			
Ao Peak Velocity (mm·s^−1^)	1242.40 ± 31.94	1040.70 ± 26.73^*^	1006.70 ± 76.22^*^
Ao Peak Gradient (mmHg)	6.20 ± 0.32	4.36 ± 0.22^*^	4.16 ± 0.33^*^
Ao Mean Gradient (mmHg)	1.79 ± 0.11	1.27 ± 0.08	1.21 ± 0.77
Ao Velocity Time Integral (cm)	4.15 ± 0.17	3.62 ± 0.20	3.34 ± 0.25^*^

Data are presented as mean ± SE, *n* = 5 mice in each group. *P < 0.05 compared to WT and ^#^*P* < 0.05 compared between cTnT‐ΔΕ7 and ΔΕ7+eTnT groups using Student's *t* test.

**Figure 3.**
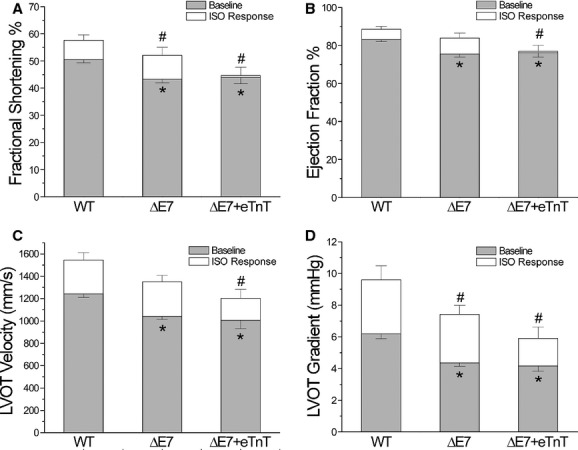
Effects of isoproterenol on cardiac function in vivo. (A and B) Baseline fraction shortening (FS) and ejection fraction (EF) were significantly decreased in cTnT‐ΔΕ7 and ΔΕ7+eTnT mouse hearts as compared to WT controls. Upon isoproterenol (ISO) stimulation, FS and EF in WT and cTnT‐ΔΕ7 mouse hearts were increased, whereas ΔΕ7+eTnT hearts had no significant response to ISO stimulation. (C and D) Aortic Doppler data demonstrated that left ventricular outflow track (LVOT) velocity and gradient were decreased significantly in cTnT‐ΔΕ7 and ΔΕ7+eTnT mice compared to that of WT mice. ISO produced increases in all groups, whereas the levels remained lower in cTnT‐ΔΕ7 and ΔΕ7+eTnT hearts than that of WT control. The values are mean ± SD. *n* = 5 in each group. *P < 0.05, compared to WT at baseline and ^#^*P* < 0.05, compared to WT upon ISO treatment, in Student's *t* test.

The results in Fig. 3A and B showed that LV fractional shortening (FS) and ejection fraction (EF), the most commonly used indexes of global LV systolic function, measured using echocardiography were both decreased in cTnT‐ΔΕ7 and ΔΕ7+eTnT transgenic mouse hearts as compared to WT control. Isoproterenol stimulation significantly increased FS and EF of WT and cTnT‐ΔΕ7 hearts, whereas the response was minimum in ΔΕ7+eTnT hearts (Fig. 3A and B). Whereas the beta‐adrenergic response of pump function (FS and EF) was blunted, contractile velocity was unchanged ΔΕ7+eTnT hearts (Table 1). While the molecular mechanism remains to be investigated, this phenotype is consistent with the more severely decreased stroke volume (Fig. 9E) and cardiac efficiency (Fig. 10B) of the ΔΕ7+eTnT hearts.

No significant change was found in mitral Doppler and tissue Doppler measurements (Table 1), except that left ventricular contraction time (LVCT) was increased in ΔΕ7+eTnT mice as compared to that of WT and cTnT‐ΔΕ7 mice, suggesting a lower LV contractile velocity in the ΔΕ7+eTnT hearts. In contrast, left ventricular relaxation time (LVRT) did not show significant change in the transgenic mouse hearts.

To evaluate the kinetic function of the heart, left ventricular outflow tract (LVOT) velocity was measured from the apical approach in an anteriorly angulated four‐chamber view. Aortic Doppler data indicated that LVOT velocity and LVOT gradient were decreased in cTnT‐ΔΕ7 and ΔΕ7+eTnT hearts as compared to WT control (Fig. 3C and D). LVOT velocity and gradient were both flow dependent. The results indicate less flow at aortic valves during systole resulting from reduced systolic function in cTnT‐ΔΕ7 and ΔΕ7+eTnT mouse hearts. ΔΕ7+eTnT hearts showed less response of LVOT velocity and LVOT gradient to isoproterenol stimulation in comparison with that of WT and cTnT‐ΔΕ7 hearts (Fig. 3C and D).

### No change in phosphorylation of cardiac TnI and myosin‐binding protein C in vivo

**Figure 4.**
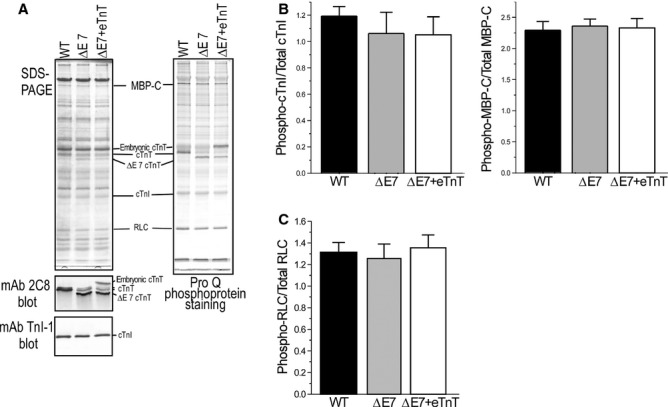
*β*‐adrenergic‐dependent phosphorylation of cardiac TnI and MBP‐C was preserved. (A) Normalized to the level of actin, SDS‐PAGE gel and Pro‐Q staining showed no significant difference in the phosphorylation levels of cardiac TnI and MBP‐C in WT, cTnT‐ΔE7 and ΔE7+eTnT mouse hearts in vivo under isoproterenol (ISO) stimulation. mAb 2C8 Western blot confirmed the expression of cTnT‐ΔE7 and eTnT in the transgenic mouse hearts. mAb TnI‐1 Western blot showed similar levels of cardiac TnI in all three groups. (B) Densitometry quantification showed no statistical difference among the three groups. (C) There was also no difference in the phosphorylation of myosin regulatory light chain among the three groups. The values are mean ± SE. *n* = 5 mice in each group. Statistical analysis was done using one‐way ANOVA.

Pro‐Q diamond phosphoprotein staining of SDS‐PAGE gels examined the phosphorylation level of cardiac TnI and myosin‐binding protein C in mouse hearts under isoproterenol treatment in vivo (Fig. 4). The results did not find significant difference among the three groups, suggesting that *β*‐adrenergic signaling was preserved and the impaired systolic function may indicate direct effects of cTnT‐ΔΕ7 or ΔΕ7+eTnT on cardiac muscle contractility. Representing other phosphorylation‐regulated myofilament proteins, the phosphorylation level of myosin regulatory light chain (RLC) also had no significant change in the cTnT‐ΔΕ7 and ΔΕ7+eTnT mouse hearts (Fig. 4C).

### cTnT‐ΔΕ7 and ΔΕ7+eTnT decreased systolic function of ex vivo working hearts

**Figure 5.**
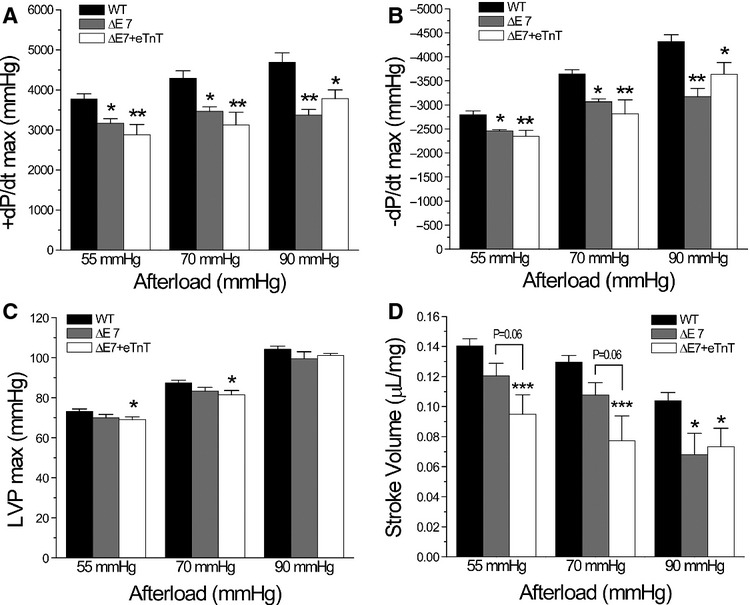
Working heart performance at different afterloads. (A and B) +dP/dt_max_ and –dP/dt_max_ were increased when afterload was increased from 55 mmHg to 90 mmHg in WT hearts. +dP/dt_max_ and –dP/dt_max_ were significantly slower in cTnT‐ΔΕ7 and ΔΕ7+eTnT hearts than WT controls with minimum responses to the increases in afterload. (C) LVP_max_ increased in all three groups in response to increases in afterload. ΔΕ7+eTnT hearts produced lower LVP_max_ than that of WT hearts at 55 mmHg and 70 mmHg afterloads. (D) Stroke volume decreased in response to increases in afterload in all groups. cTnT‐ΔΕ7, and more obviously ΔΕ7+eTnT hearts, had significantly lower stroke volume than that of WT hearts at all afterloads tested. *n* = 10 in WT, *n* = 5 in cTnT‐ΔΕ7 and *n* = 6 in ΔΕ7+eTnT groups. The values are mean ± SE. *P < 0.05 and **P < 0.01 versus WT groups in one‐way ANOVA.

To exclude the effects of neurohumoral and vascular compensation on cardiac function in vivo, isolated ex vivo working heart preparations under precisely controlled preload, afterload and heart rate provide further information for the effects of cTnT‐ΔΕ7 or ΔΕ7+eTnT on the function of cardiac muscle. In addition to baseline measurements at preload of 10 mmHg and afterload of 55 mmHg, 70 mmHg, and 90 mmHg were used to apply afterload stress. The results showed that in WT hearts, ±dP/dt and maximum LVP (LVP_max_) increased at higher afterloads, whereas stoke volume decreased in response to the increase in afterload (Fig. 5).

cTnT‐ΔΕ7 and ΔΕ7+eTnT hearts had significantly slower systolic and diastolic velocities (Fig. 5A and B), LVP_max_ (Fig. 5C), and stroke volume (Fig. 5D) than that of WT hearts at baseline and more severe at higher afterloads. The diastolic LVP (LVP_min_) was unchanged (data no shown), indicating that the end diastolic pressure and myocardial compliance did not change in the transgenic mouse hearts under normal or increased afterloads. The inability to increase ±dP/dt in cTnT‐ΔΕ7 and ΔΕ7+eTnT hearts when afterload was increased suggests a diminished contractile capacity.

**Figure 6.**
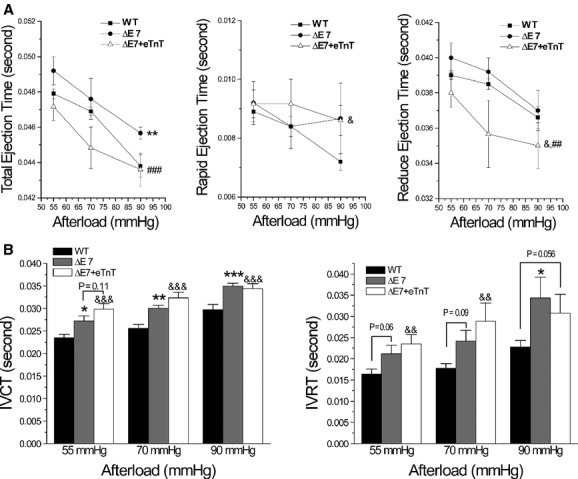
Time parameters of working heart at different afterloads. (A) Similar trends of decrease in total and reduced ejection time were seen in cTnT‐ΔΕ7, ΔΕ7+eTnT and WT hearts when afterload was increased from 55 to 90 mmHg. Total and reduced ejection times were longer in cTnT‐ΔΕ7 hearts but shorter in ΔΕ7+eTnT hearts as compared to that of WT hearts. Rapid ejection time was longer in cTnT‐ΔΕ7 and ΔΕ7+eTnT hearts than WT control at 90 mmHg afterload. (B) Indicating slower initial systolic and diastolic velocities, IVCT, and IVRT increased in all three groups when afterload was increased and were longer in cTnT‐ΔΕ7 and ΔΕ7+eTnT hearts than that of WT hearts. *n* = 10 in WT, *n* = 5 in cTnT‐ΔΕ7 and *n* = 6 in ΔΕ7+eTnT groups. The values are mean ± SE. *P < 0.05, **P < 0.01, and ***P < 0.001 cTnT‐ΔΕ7 versus WT; ^&^*P* < 0.05 and ^&&&^*P* < 0.001 ΔΕ7+eTnT versus WT; ^##^*P* < 0.01 and ^###^*P* < 0.001 ΔΕ7+eTnT versus cTnT‐ΔΕ7. Statistical tests were performed using two‐way ANOVA for panel A and one‐way ANOVA for panel B.

When afterload was increased, the time parameters of WT mouse hearts showed decreased total, rapid, and reduced ejection times (Fig. 6A), corresponding to the reduced stroke volume (Fig. 5D). Total ejection time was prolonged in cTnT‐ΔΕ7 hearts and reduced ejection time was shortened in ΔΕ7+eTnT hearts. There were similar decreases in total and reduced ejection time of cTnT‐ΔΕ7 and ΔΕ7+eTnT hearts when afterload was increased. At 90 mmHg rapid ejection time did not shorten significantly in cTnT‐ΔΕ7 and ΔΕ7+eTnT hearts when afterload was increased, therefore, was longer than that of WT (Fig. 6A).

Isovolumetric contraction and relaxation times (IVCT and IVRT) were longer in cTnT‐ΔΕ7 and ΔΕ7+eTnT hearts than WT controls at all afterloads tested (Fig. 6B), indicating slower initial systolic and diastolic velocities (Fig. 5A and B) in comparison to that of WT hearts.

### Decreased efficiency of cTnT‐ΔΕ7 and ΔΕ7+eTnT hearts

**Figure 7.**
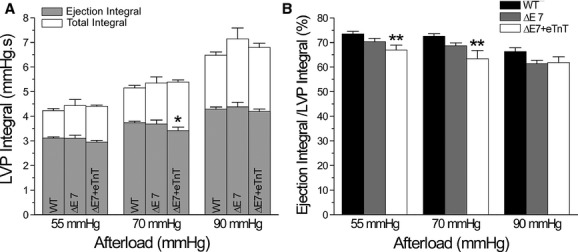
Cardiac efficiency indicated by LVP integrals. (A) Ejection integral was lower in ΔΕ7+eTnT hearts than that of WT hearts at 70 mmHg afterload. (B) The ratio of ejection integral versus total LVP integral was lower in ΔΕ7+eTnT hearts at 55 and 70 mmHg afterload than WT control. *n* = 10 in WT, *n* = 5 in cTnT‐ΔΕ7 and *n* = 6 in ΔΕ7+eTnT groups. The values are mean ± SE. *P < 0.05 and **P < 0.01 versus WT in one‐way ANOVA.

LVP integral and ejection integral were increased when afterload was increased in all three groups of hearts, in which the ejection integral had proportionally less increase indicating decreased pumping efficiency under high afterload (Fig. 7A). An increase in ejection integral reflects increased stroke work during ejection, while the increase in LVP integral corresponds to increased energy consumption. The pumping efficiency calculated from LVP ejection integral versus total integral detected that ΔΕ7+eTnT hearts had lower efficiency at 55 and 70 mmHg afterloads as compared with WT controls (Fig. 7B).

**Figure 8.**
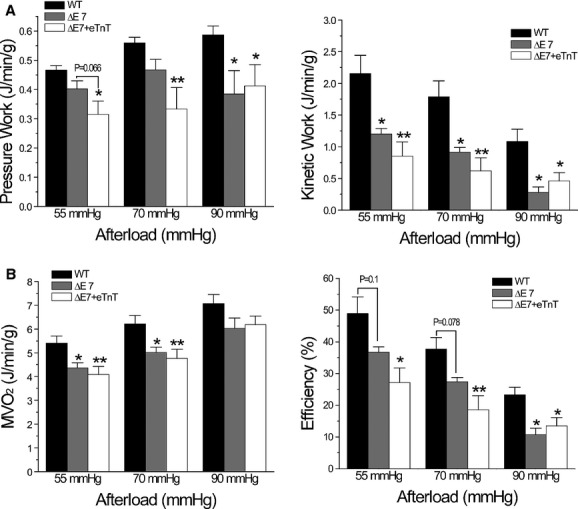
Cardiac efficiency determined from oxygen consumption. (A) WT hearts showed increased pressure work and decreased kinetic work when afterload was increased. cTnT‐ΔΕ7 and ΔΕ7+eTnT hearts produced lower pressure work and kinetic work than that of WT hearts. Increases in afterload decreased kinetic work in cTnT‐ΔΕ7 and ΔΕ7+eTnT hearts like that in WT hearts without significantly altering pressure work. (B) Oxygen consumption (MVO_2_) was increased in all three groups when afterload was increased, in which cTnT‐ΔΕ7 and ΔΕ7+eTnT hearts showed lower values than WT control. Cardiac efficiency calculated from the external cardiac work (the sum of pressure work and kinetic work) versus MVO_2_ was decreased in cTnT‐ΔΕ7 and, more severely, in ΔΕ7+eTnT hearts. Increases in afterload decreased cardiac efficiency in all three groups and augmented the difference between cTnT‐ΔΕ7 and ΔΕ7+eTnT hearts and WT control. *n* = 10 in WT, *n* = 5 in cTnT‐ΔΕ7 and *n* = 6 in ΔΕ7+eTnT groups. The values are mean ± SE. *P < 0.05 and **P < 0.01 versus WT. in two‐way ANOVA.

To further investigate the decreased cardiac efficiency using the classic approach of measuring cardiac output versus oxygen consumption, the results showed that pressure work was lower in cTnT‐ΔΕ7 and ΔΕ7+eTnT hearts than that in WT hearts at all afterloads tested (Fig. 8A). Pressure work was increased in WT but not cTnT‐ΔΕ7 and ΔΕ7+eTnT hearts when afterload was increased (Fig. 8A). Kinetic work was decreased (Fig. 8A) and MVO_2_ increased in all three groups of hearts when afterload was increased. These changes resulted in a reduction of energetic efficiency that was more severe in cTnT‐ΔΕ7 and ΔΕ7+eTnT hearts than that in WT hearts and diminished more at higher afterload in all three groups (Fig. 8B). It was worth noting that although MVO_2_ was lower in cTnT‐ΔΕ7 and ΔΕ7+eTnT hearts than that of WT hearts (Fig. 8B), the ratio of cardiac work versus energy expenditure was significantly lower in cTnT‐ΔΕ7 and ΔΕ7+eTnT hearts due to drastically decreased work, corresponding to lower energetic efficiency (Fig. 8B).

### Preserved response to preload in cTnT‐ΔΕ7 and ΔΕ7+eTnT hearts

**Figure 9.**
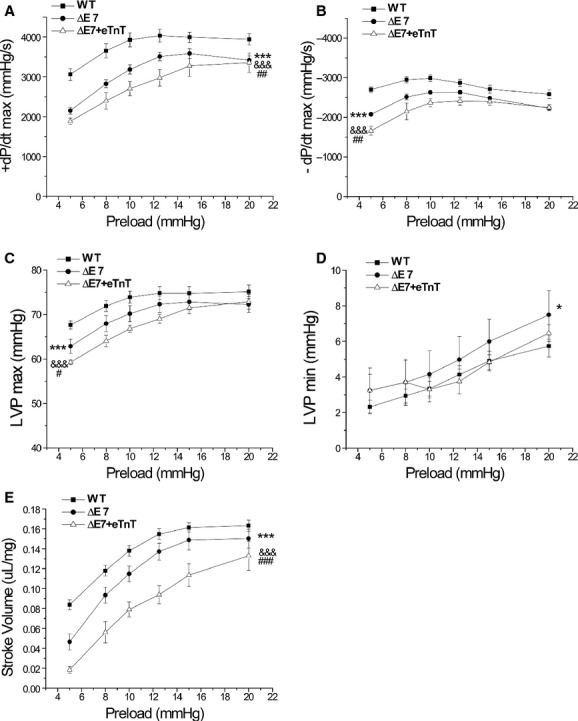
Response of ex vivo working hearts to changes in preload. (A and B) Systolic (+dP/dt_max_) and diastolic (‐dP/dt_max_) velocities of cTnT‐ΔΕ7 and ΔΕ7+eTnT hearts were significantly slower than that of WT hearts at all preloads tested. Comparing to cTnT‐ΔΕ7, ΔΕ7+eTnT hearts had slower ±dP/dt_max_ at preloads from 5 to 12.5 mmHg. (C) LVP_max_ was lower in cTnT‐ΔΕ7 than that of WT hearts. ΔΕ7+eTnT hearts showed further decreases in LVP_max_ at preloads of 5 to 12.5 mmHg. (D) LVP_min_ increased in all three groups when preload was increased. cTnT‐ΔΕ7 hearts had higher LVP_min_ than that of WT hearts. (E) Stroke volume increased when preload was increased and reached to a plateau at 15 mmHg in WT and cTnT‐ΔΕ7 hearts. Stroke volume of cTnT‐ΔΕ7 hearts was significantly lower than that of WT hearts at all preloads tested. ΔΕ7+eTnT hearts had further decreased stroke volume at all preloads tested but it continued the increase at 20 mmHg preload. *n* = 10 in WT, *n* = 5 in cTnT‐ΔΕ7 and *n* = 6 in ΔΕ7+eTnT groups. Values are mean ± SE. *P < 0.05 and ***P < 0.001 cTnT‐ΔΕ7 versus WT; ^&&&^*P* < 0.001 ΔΕ7+eTnT versus WT; ^##^*P* < 0.01 and ^###^*P* < 0.001 ΔΕ7+eTnT versus cTnT‐ΔΕ7, in two‐way ANOVA.

At afterload of 55 mmHg and heart rate of 480 beats per minute, increases in preload enhanced ventricular function as measured by ±dP/dt, LVP, and stroke volume (Fig. 9), similarly in WT, cTnT‐ΔΕ7 and ΔΕ7+eTnT hearts. Although functions of cTnT‐ΔΕ7 and ΔΕ7+eTnT hearts were consistently lower than WT controls at the wide range of preload tested (Fig. 9), the results demonstrated preserved Frank‐Starling response in cTnT‐ΔΕ7 and ΔΕ7+eTnT hearts, which were capable of compensating for the impaired systolic function.

**Figure 10.**
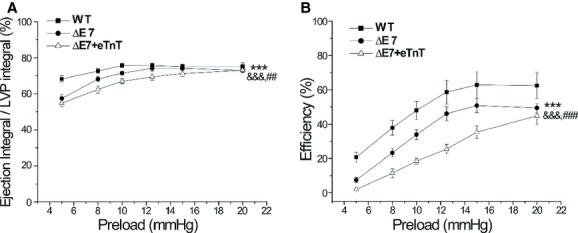
Cardiac efficiency in responses to preload. (A) Pumping efficiency calculated as the ratio of ejection integral versus LVP integral was increased when preload was increased from 5 to 10 mmHg in WT hearts. cTnT‐ΔΕ7 and ΔΕ7+eTnT hearts had lower pumping efficiency than that of WT hearts at preloads of 5 to 12.5 mmHg, which was increased to reach the WT level at high preload of 20 mmHg. (B) Cardiac efficiency calculated as the ratio of cardiac work to MVO_2_ was significantly lower in cTnT‐ΔΕ7 and ΔΕ7+eTnT hearts than that of WT hearts. Increases in preload increased the efficiencies of cTnT‐ΔΕ7 and ΔΕ7+eTnT hearts, which, however, remained lower than WT control. *n* = 10 in WT, *n* = 5 in ΔΕ7 and *n* = 6 in ΔΕ7+eTnT groups. Values are mean ± SE. ***P < 0.001 cTnT‐ΔΕ7 versus WT; ^&&&^*P* < 0.001 ΔΕ7+eTnT versus WT; ^##^*P* < 0.01 and ^###^*P* < 0.001 ΔΕ7+eTnT versus cTnT‐ΔΕ7 in two‐way ANOVA.

In WT hearts, raising preload increased both ejection integral and LVP integral (data not shown) as a result of increased contractility. Pumping efficiency deduced from the ratio of ejection integral to LVP integral increased when preload increased from 5 mmHg to 10 mmHg whereas further increases in preload did not produce significant change (Fig. 10A). cTnT‐ΔΕ7 and ΔΕ7+eTnT hearts had lower pumping efficiency, which was improved when preload was increased, reaching the level of WT control at 20 mmHg (Fig. 10A).

The effect of increasing preload on improving energetic efficiency of cTnT‐ΔΕ7 and ΔΕ7+eTnT hearts was further demonstrated by the ratio of cardiac work versus oxygen consumption (Fig. 10B). Cardiac efficiency was significantly lower in cTnT‐ΔΕ7 and ΔΕ7+eTnT hearts than that of WT hearts at all preloads tested (Fig. 10B). Therefore, impaired systolic function appeared to be a determining factor in reducing myocardial energetic efficiency. However, increases in preload did improve energetic efficiency of cTnT‐ΔΕ7 and ΔΕ7+eTnT hearts (Fig. 10B). The results confirmed the effect of increasing preload on compensating for the impaired systolic function of cTnT‐ΔΕ7 and ΔΕ7+eTnT hearts.

## Discussion

cTnT‐ΔΕ7 is an aberrant splicing variant found in turkey and dog dilated cardiomyopathies (Biesiadecki and Jin 2002; Biesiadecki et al. 2002). The embryonic splice form of cardiac TnT normally expresses in embryonic and neonatal heart and skeletal muscle (Jin 1996). Its expression in adult heart is found coexisting with cTnT‐ΔΕ7 in dogs with dilated cardiomyopathy (Biesiadecki et al. 2002) as represented by the ΔΕ7+eTnT double‐transgenic mice. Extended from previous studies, the present work demonstrated the pathophysiology of cTnT‐ΔΕ7 and ΔΕ7+eTnT in transgenic mouse models in vivo and ex vivo with the following new findings.

### Effects of cardiac TnT N‐terminal abnormality on systolic function of the heart

Quantitative studies on multiple functional parameters demonstrated that cTnT‐ΔΕ7 and cTnT‐ΔΕ7+eTnT hearts had specifically decreased systolic function both in vivo and in ex vivo working heart preparations. cTnT‐ΔΕ7 and eTnT differ from wild‐type adult cardiac TnT in the N‐terminal region (Biesiadecki et al. 2002). The N‐terminal region of TnT is a variable structure that differs among muscle type‐specific isoforms and is regulated by alternative splicing during heart and muscle development and adaptation (Wei and Jin 2011). In previous studies, we and others have demonstrated the function of the N‐terminal variable region in regulating myofilament Ca^2+^ sensitivity (Biesiadecki and Jin 2002; Gomes et al. 2004; Mamidi et al. 2013a) and interaction with tropomyosin. An overall observation is that a longer N‐terminal segment with more negatively charged residues produces higher Ca^2+^ sensitivity (Reiser et al. 1992, 1996; Ogut et al. 1999; Mamidi et al. 2013b).

Consistent with the nature of the N‐terminal segment of TnT as a regulatory structure, N‐terminal abnormal splicing has been detected in failing human hearts (Anderson et al. 1995; Mesnard‐Rouiller et al. 1997). Another example for the regulatory function of the N‐terminal segment of cardiac TnT is its selective removal by restrictive proteolysis in adaptation to ischemia‐reperfusion or pressure overload (Zhang et al. 2006; Feng et al. 2008). Overexpression of the N‐terminal truncated cTnT resulted in decreased contractile velocity in transgenic mice (Feng et al. 2008), supporting the notion that modification in the N‐terminal region of cardiac TnT regulates systolic function of the heart.

### Impaired systolic function and TnT heterogeneity decrease cardiac efficiency

The ventricular ejection time is a crucial parameter in determining cardiac output and energetic efficiency (Braunwald et al. 1958; Sarnoff et al. 1958; Weissler et al. 1968; Lewis et al. 1977; Geeraerts et al. 2004; Gutterman and Cowley 2006; Feng and Jin 2010). The N‐terminal variation of cardiac TnT plays a role on regulating the time of ventricular ejection. For example, N‐terminal truncated cardiac TnT prolongs the rapid ejection phase by moderately reducing systolic velocity without decreasing LVP_max_, which increases cardiac efficiency (Feng et al. 2008). Similarly, Fig. 6A showed that cTnT‐ΔΕ7 and ΔΕ7+eTnT hearts had prolonged rapid ejection phase, especially at higher afterload (90 mm Hg).

However, the compensatory effect of prolonged ejection time of cTnT‐ΔΕ7 and ΔΕ7+eTnT hearts did not completely correct the decreased cardiac efficiency due to their effects on decreasing LVP_max_ that severely reduced systolic function. In addition, ΔΕ7+eTnT hearts had even shorter total and reduced ejection times than that of WT hearts, reflecting worse systolic function. We previously demonstrated that TnT heterogeneity, i.e., the coexistence of more than one class of TnT in the cardiac myofilaments, decreased heart function and energetic efficiency by desynchronizing myofilaments' response to the rising and decaying of cytosolic Ca^2+^ (Huang et al. 2008; Yu et al. 2012). IVCT and IVRT that reflect nonwork energy consumption were prolonged in cTnT‐ΔΕ7 and more in ΔΕ7+eTnT hearts (Fig. 6B) due to decreased systolic and diastolic velocities, consistent with desynchronized myofilament actions and decreased energetic efficiency from increasing nonwork energy consumption. These dominantly negative effects could explain the decreased efficiency in cTnT‐ΔΕ7 hearts and more obviously in ΔΕ7+eTnT hearts.

The mechanisms by which the N‐terminal variable region of cardiac TnT affects Ca^2+^ sensitivity and systolic function of the heart require further study. Results from this line of investigation may identify a therapeutic range of decreasing myofilament Ca^2+^ sensitivity to prolong rapid ejection time without significant decrease in force development and LVP_max_.

### Preserved Frank‐Starling response partially compensate for the impaired systolic function of cTnT‐ΔΕ7 and ΔΕ7+eTnT hearts

Indicated by the similar left ventricular end diastolic dimension seen in echocardiographs (Table 1) and LVP_min_ in response of afterloads (data not shown), the young cTnT‐ΔΕ7 and ΔΕ7+eTnT transgenic mouse hearts were at a compensated stage without anatomical dilation or clinical failure.

The results in Fig. 9 showed preserved Frank‐Starling response of cTnT‐ΔΕ7 and ΔΕ7+eTnT hearts, demonstrating that the N‐terminal abnormality in cardiac TnT does not abolish the Frank‐Starling regulation of cardiac muscle. Diastolic function of the ventricular muscle is one of the key factors that determine the Frank‐Starling response of the heart, which may be separated from the negative effect of cardiac TnT N‐terminal abnormality on systolic function.

Nonetheless, a chronic adaptive utilization of Frank‐Starling mechanism to compensate for the impaired systolic function would increase energy expenditure and induce dilatative ventricular remodeling, leading to the progression of dilated cardiomyopathy in cTnT‐ΔΕ7 and ΔΕ7+eTnT hearts (Yu et al. 2012).

### Potential benefit when eTnT is expressed together with cTnT‐ΔΕ7

While cTnT‐ΔΕ7 is an abnormally spliced mutant form of cardiac TnT, eTnT is a normal TnT naturally expressed in embryonic hearts (Jin and Lin 1989). cTnT‐ΔΕ7 was found in both turkey and dog dilated cardiomyopathies as a primary pathogenic abnormality. While the dominantly negative impact and pathogenic effects of exon 7 deletion in cardiac TnT have been demonstrated (Wei et al. 2010), the effect of the expression of embryonic cardiac TnT in adult heart on the development of dilated cardiomyopathy is worth investigating.

The contractility data in Fig. 5A showed that while cTnT‐ΔΕ7 hearts failed to have positive inotropic responses when afterload increased from 70 mmHg to 90 mmHg, ΔΕ7+eTnT hearts maintained stroke volume. This observation implicates that the coexistence of eTnT might contribute a compensation for the impaired systolic function from the primarily pathogenic cTnT‐ΔΕ7.

Nonetheless, the presence of an additional class of TnT in ΔΕ7+eTnT double‐transgenic mouse hearts increases myofilament heterogeneity that would decrease cardiac efficiency (Feng and Jin 2010). The in vivo cardiac function (Fig. 3A and B) showed that in contrast to that of cTnT‐ΔΕ7 hearts, ΔΕ7+eTnT hearts had diminished kinetic response to isoproterenol stimulation. The preload responses shown in Fig. 9 also indicated that ΔΕ7+eTnT hearts had the lowest inotropic function at multiple levels of preloads. Therefore, the potential benefit of coexistence of embryonic cardiac TnT to compensate for the impaired function of cTnT‐ΔΕ7 hearts is limited by the negative effect on reducing cardiac efficiency.

## Acknowledgments

We thank Dr. M.M. Hossain for maintaining the transgenic mouse lines and Hui Wang for mouse genotyping.

## Conflict of Interest

No competing financial interests are declared by the author(s).
